# Federated Learning for Histopathology Image Classification: A Systematic Review

**DOI:** 10.3390/diagnostics16010137

**Published:** 2026-01-01

**Authors:** Meriem Touhami, Mohammad Faizal Ahmad Fauzi, Zaka Ur Rehman, Sarina Mansor

**Affiliations:** 1 Faculty of AI and Engineering, Multimedia University, Persiaran Multimedia, Cyberjava 63100, Malaysia; 1231400795@student.mmu.edu.my; 2 School of Digital Health, KPJ Healthcare University, Nilai 71800, Malaysia; faizal.fauzi@kpju.edu.my; 3 Centre for Image and Vision Computing, COE for Artificial Intelligence, Multimedia University, Cyberjaya 63100, Malaysia

**Keywords:** federated learning, medical image classification, disease diagnosis, data privacy, deep learning, machine learning, non-IID data, model aggregation, systematic literature review

## Abstract

**Background/Objective**: The integration of machine learning (ML) and deep learning (DL) has significantly enhanced medical image classification, especially in histopathology, by improving diagnostic accuracy and aiding clinical decision making. However, data privacy concerns and restrictions on sharing patient data limit the development of effective DL models. Federated learning (FL) offers a promising solution by enabling collaborative model training across institutions without exposing sensitive data. This systematic review aims to comprehensively evaluate the current state of FL applications in histopathological image classification by identifying prevailing methodologies, datasets, and performance metrics and highlighting existing challenges and future research directions. **Methods**: Following PRISMA guidelines, 24 studies published between 2020 and 2025 were analyzed. The literature was retrieved from ScienceDirect, IEEE Xplore, MDPI, Springer Nature Link, PubMed, and arXiv. Eligible studies focused on FL-based deep learning models for histopathology image classification with reported performance metrics. Studies unrelated to FL in histopathology or lacking accessible full texts were excluded. **Results**: The included studies utilized 10 datasets (8 public, 1 private, and 1 unspecified) and reported classification accuracies ranging from 69.37% to 99.72%. FedAvg was the most commonly used aggregation algorithm (14 studies), followed by FedProx, FedDropoutAvg, and custom approaches. Only two studies reported their FL frameworks (Flower and OpenFL). Frequently employed model architectures included VGG, ResNet, DenseNet, and EfficientNet. Performance was typically evaluated using accuracy, precision, recall, and F1-score. Federated learning demonstrates strong potential for privacy-preserving digital pathology applications. However, key challenges remain, including communication overhead, computational demands, and inconsistent reporting standards. Addressing these issues is essential for broader clinical adoption. **Conclusions**: Future work should prioritize standardized evaluation protocols, efficient aggregation methods, model personalization, robustness, and interpretability, with validation across multi-institutional clinical environments to fully realize the benefits of FL in histopathological image classification.

## 1. Introduction

**Figure 1:**
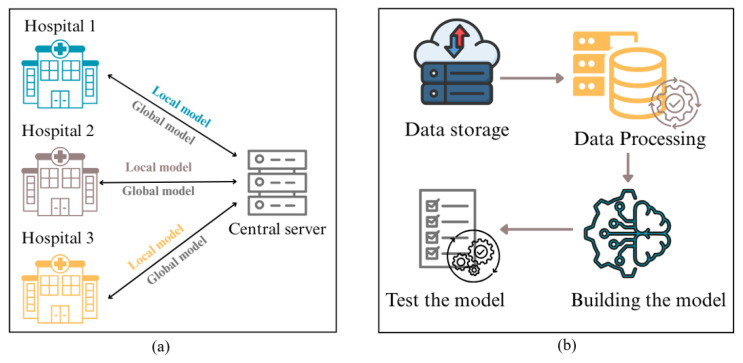
Comparison of (**a**) federated learning (FL) workflow and (**b**) traditional machine learning (ML) workflow.

In recent years, deep learning techniques have demonstrated exceptional predictive capabilities across various fields, including computational pathology [1,2]. However, their effectiveness is highly dependent on access to large and diverse datasets, a requirement that poses a substantial challenge in healthcare [3]. In contrast to other fields where data is readily available, medical datasets are difficult to obtain due to strict privacy regulations, ethical considerations, and logistical challenges in data collection [4]. Healthcare institutions are bound by stringent regulations such as the Health Insurance Portability and Accountability Act (HIPAA) in the United States and the General Data Protection Regulation (GDPR) in Europe [5]. These regulations place rigorous restrictions on the sharing, storage, and processing of patient data. These laws are intended to protect sensitive medical information, thereby ensuring the consistent preservation of patient confidentiality. However, this gives rise to a fundamental conflict between the need to safeguard individual privacy and the necessity of aggregating sufficient data to build accurate, reliable deep learning models for disease classification and diagnosis. Traditional machine learning approaches rely on centralized data collection, where medical institutions must transfer patient data to a shared repository for model training. Given the legal constraints and the understandable reluctance of healthcare providers to share confidential patient information, a centralized data-collection approach is largely impractical for large-scale medical applications [6]. To tackle these challenges, federated learning (FL) has emerged as a powerful and privacy-preserving solution, enabling institutions to jointly train machine learning models without directly sharing their raw data [7,8]. Figure 1 illustrates the comparison between traditional machine learning workflow and the federated learning workflow, highlighting the key differences in data handling and model training processes.

Federated learning (FL) was first introduced by H. Brendan McMahan et al in 2016–2017 as a privacy-preserving, distributed machine learning approach for mobile devices, notably demonstrated in applications such as next word prediction and query suggestions for the Gboard keyboard [8]. In [9], the authors showed that using the Federated Averaging algorithm on device-generated data improved prediction recall while keeping user data localized. Similarly, Ref. [10] applied federated learning at a global scale to enhance keyboard query suggestions without accessing user typing data. Recognizing the privacy and collaborative advantages of federated learning, from 2018 to 2021, researchers began adapting the approach to healthcare, leveraging its ability to integrate knowledge from multiple hospitals and research centers while complying with data protection regulations.

Alternatively, rather then requiring hospitals and research centers to transfer sensitive patient images to a central server, federated learning allows each institution to train a local model using its own data stored securely within its network [11]. Once local training is completed, only the model updates, such as gradients or weights, are shared with a central aggregation server, which then integrates these updates from multiple sites to create a globally improved model [12]. Since no raw data ever leaves the premises of the participating institutions, federated learning ensures compliance with HIPAA and GDPR regulations, addressing privacy concerns while still allowing for effective machine learning development [13]. This decentralized approach effectively eliminates the risks associated with data breaches and unauthorized access, making it a viable strategy for medical fields where patient confidentiality is paramount.

Federated learning has proven particularly valuable in histopathology image classification, where datasets often suffer from data scarcity, heterogeneity, and imbalance due to differences in imaging techniques, staining protocols, and patient demographics across institutions [14,15]. Overall, federated learning (FL) addresses critical challenges in histopathology image classification by enabling collaborative model training across diverse datasets while maintaining privacy and security.

However, despite growing interest in federated learning (FL) for medical imaging, most reviews focus on general tasks or specific modalities, leaving histopathology largely unexplored. This field faces unique challenges, including gigapixel-scale whole-slide images, severe class imbalance, and variability in staining and hardware, yet lacks a comprehensive review analyzing federated learning applications, datasets, frameworks, and strategies for handling non-IID data and privacy. Such an analysis is essential to evaluate federated learning’s readiness for clinical use and to identify existing methodological gaps.

**Figure 2:**
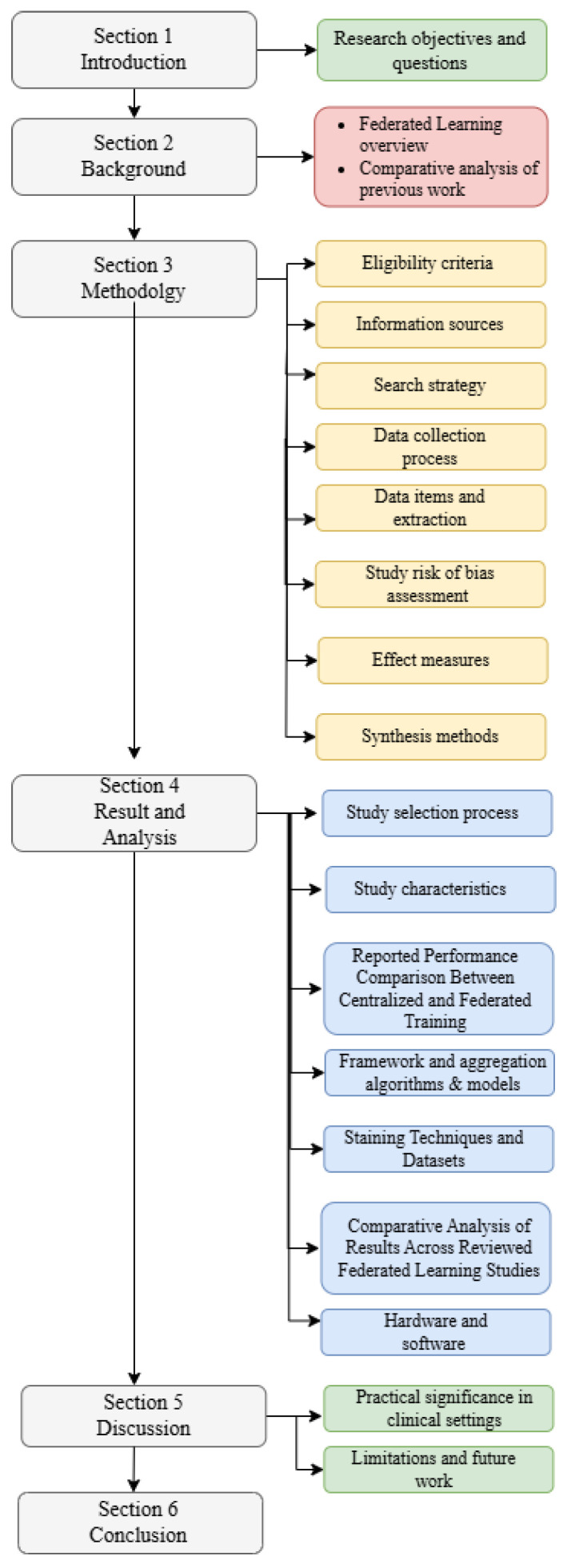
Overall structure of this systematic literature review on federated learning for histopathology image classification.

The remainder of this paper is structured as follows. Section 2 introduces the background of the study, including an overview of federated learning (FL) and related work, highlighting existing surveys in the field. Section 3 outlines the methodology, detailing search strategy, inclusion and exclusion criteria, data extraction, and study selection process. Section 4 presents the results, providing answers to the research questions. Section 5 discusses the limitations identified in the review and suggests future research directions. Finally, Section 6 concludes the paper. The overall structure of this paper is presented in Figure 2.

### Research Objectives and Questions

The primary objective of this systematic review is to systematically evaluate how federated learning has been applied to histopathology image classification, with a focus on its effectiveness in enabling collaborative model training without compromising patient data privacy. A secondary objective of this study is to provide a detailed analysis of the FL architectures, algorithms, and model designs used in digital pathology, while also investigating the extent to which dataset heterogeneity, particularly differences across medical institutions, impacts the performance of federated learning. Additionally, this study aims to identify current limitations, challenges, and gaps in the existing research, and to outline future directions and opportunities for advancing the adoption of federated learning in digital pathology. To achieve these objectives, this review adopts a question-driven approach designed to systematically extract, organize, and synthesize key insights from the existing body of literature. These research questions collectively guide the investigation by addressing different dimensions of FL application in histopathology image classification:

1. How effective is federated learning in improving histopathology image classification performance while addressing data privacy and sharing limitations?
2. Which datasets and staining techniques are commonly used in federated learning for histopathology image classification?
3. What federated learning frameworks, aggregation algorithms, and classification models are employed in histopathology image analysis?
4. How do different federated learning approaches compare in terms of classification performance for histopathology images?
5. What software frameworks and hardware infrastructures are used in implementing federated learning for cancer histopathology image classification?

These questions aim to identify existing research gaps and propose potential pathways for improving privacy-preserving, scalable, and interpretable FL solutions in histopathology.

## 2. Background

### 2.1. Federated Learning Overview

Federated learning (FL) is a decentralized machine learning paradigm that enables multiple clients, such as hospitals or imaging centers, to collaboratively train a shared model without exchanging raw data, thereby preserving privacy [16]. Model training occurs locally at each client using its respective dataset, with only the resulting model updates being communicated to a central server. The server aggregates these updates, commonly through techniques like Federated Averaging (FedAvg) [17], to produce an improved global model, which is then redistributed to the clients. This iterative process continues until the model converges, ensuring both performance and data confidentiality.

The typical federated learning (FL) framework comprises three main components:

- **Clients (participants):** Entities holding local datasets, often non-independent and identically distributed (non-IID). In healthcare, these clients may include hospitals, clinics, or medical imaging devices operating in either cross-silo (institutional) or cross-device (individual) scenarios [18].
- **Central Server:** Coordinates the FL process by distributing the global model, collecting client updates, and updating the global model iteratively. The server can be deployed within secure environments to enhance privacy [19].
- **Communication Protocol:** Ensures encrypted transmission of model updates between clients and the central server, safeguarding against security threats during communication [20].

The FL training procedure involves iterative steps to optimize the global model [21]:

1. **Global Model Initialization:** The central server initializes the global model parameters $W^{\left(0\right)}$ and sends them to all clients at the start of each communication round (r).
2. **Local Model Training:** Each client *x* trains the received global model on its local dataset $D_{x}$ for *E* epochs. The local parameters are updated according to the following equation:
  (1) $$W_{x}^{\left(r+1\right)}\leftarrow W^{\left(r\right)}-\eta \nabla F_{x}\left(W^{\left(r\right)}\right)$$
  where η is the learning rate, $\nabla F_{x}\left(W^{\left(r\right)}\right)$ is the gradient of the local loss, and $W_{x}^{\left(r+1\right)}$ are the updated local parameters.
3. **Model Aggregation:** Clients transmit their updated parameters $W_{x}^{\left(r+1\right)}$ to the server, which aggregates them using **Federated Averaging (FedAvg)**:
  (2) $$W^{\left(r+1\right)}\leftarrow \sum_{x=1}^{X}\frac{|D_{x}|}{\left|D\right|}W_{x}^{\left(r+1\right)}$$
  Weighted averaging ensures clients with larger datasets have proportionally greater impact on the global model.
4. **Model Redistribution:** The server sends the updated global model $W^{\left(r+1\right)}$ back to all clients. This process repeats until convergence, either when performance metrics meet a threshold or after a set number of communication rounds.

**Figure 3:**
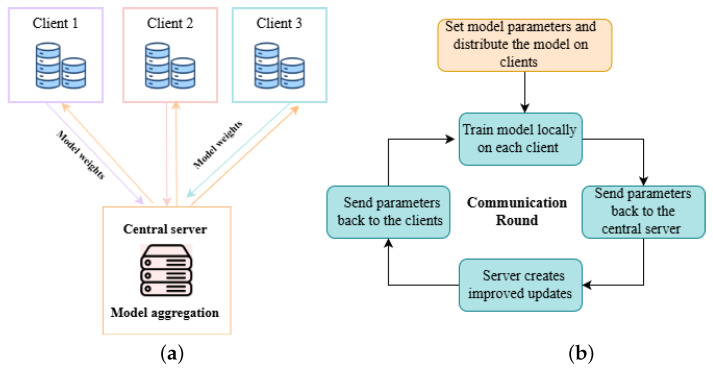
(**a**) Client–server parameter exchange in federated learning and (**b**) illustration of a communication round in a federated learning system.

Figure 3 illustrates the client–server parameter exchange in federated learning and the communication process during a training round.

#### 2.1.1. Mathematical Foundations of Federated Learning

Consider a federated learning system comprising *X* clients, where each client *x* (with x∈{1,2,…,X}) possesses a local dataset $D_{x}$. The primary goal of federated learning is to optimize a global model parameter W by minimizing the aggregated local loss functions across all participating clients. The local loss function for a given client *x* can be formulated as follows:

(3) $$F_{x}\left(W\right)=\frac{1}{|D_{x}|}\sum_{\left(p_{i},q_{i}\right)\in D_{x}}\ell \left(W;p_{i},q_{i}\right)$$

where $\ell (W;p_{i},q_{i})$ represents the loss incurred by the model with parameters W for the input feature $p_{i}$ and its corresponding label $q_{i}$. This function quantifies the prediction error of the model on client *x*’s local dataset.

The central server’s objective is to minimize the **global loss function** F(W), which is defined as the weighted sum of the local loss functions from all clients:

(4) $$F\left(W\right)=\sum_{x=1}^{X}\frac{|D_{x}|}{\left|D\right|}F_{x}\left(W\right)$$

In this equation

- $|D_{x}|$ denotes the number of samples in client *x*’s dataset.
- $\left|D\right|=\sum_{x=1}^{X}\left|D_{x}\right|$ is the total number of data points across all clients.
- The weighting factor $\frac{|D_{x}|}{\left|D\right|}$ ensures that clients with larger datasets contribute proportionally more to the global model.

#### 2.1.2. Federated Learning Approaches

**Figure 4:**
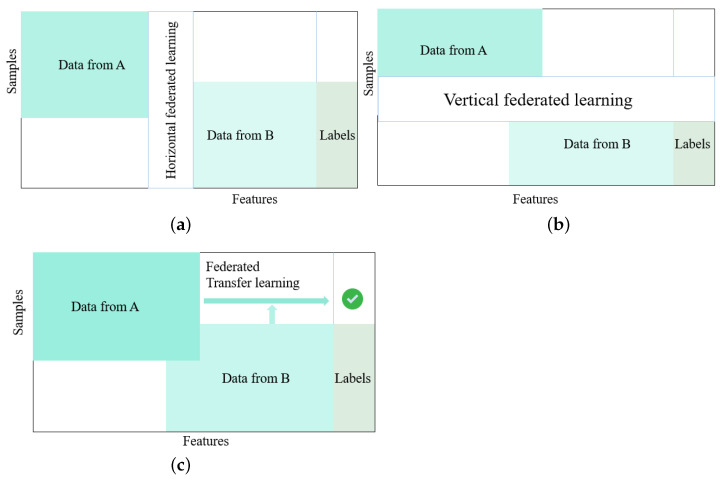
(**a**) Client–server parameter exchange in federated learning; (**b**) illustration of a communication round in a federated learning system; and (**c**) federated transfer learning approach.

In federated learning, data partitioning shapes the training strategy and results in three main approaches: horizontal FL (HFL), vertical FL (VFL), and federated transfer learning (FTL). HFL (Figure 4 presents the horizontal FL approach) is used when clients hold different samples but share the same feature space, enabling large-scale collaboration among numerous institutions or devices while maintaining privacy [22,23]. VFL (Figure 4 presents the vertical federated learning approach) applies when organizations share the same entities but collect different types of features, making it suitable for combining complementary data across domains and typically involving fewer participants with added encryption for privacy [22,23]. FTL (Figure 4 shows the federated transfer approach) addresses cases where datasets differ in both samples and feature spaces. It leverages transfer learning to enable collaboration between highly heterogeneous institutions and supports cross-domain medical applications [22,23]. Together, these approaches allow flexible and privacy-preserving collaboration across varied data distributions.

### 2.2. Related Work

Several recent systematic reviews have examined the application of federated learning (FL) in healthcare and medical imaging, but none focus specifically on histopathology image classification. For example, ref. [24] surveys FL across healthcare broadly, emphasizing data partitioning, data distribution, and application domains such as COVID-19 imaging; however, histopathology classification is barely addressed. Ref. [25] includes 612 articles and reports that only 5.2% of studies are real-life applications; radiology dominates among data modalities, with limited attention to pathology/histology. Ref. [11] reviews methods for classification and segmentation in medical imaging generally, but does not drill in on histopathology tile-based classification or center heterogeneity in histopathology image classification tasks. Ref. [26] focuses on challenges (e.g., heterogeneity; label quality) and solutions in medical image analysis; again, histopathological data are only peripherally addressed. Ref. [27] is another review that clusters FL applications by disease, imaging modality, and body part, offering overviews of architecture and performance vs. traditional ML, but without a dedicated section on histopathology classification tasks. Ref. [28] looks broadly at radiomics and medical image FL methods, but radiomics tends to focus more on imaging modalities like CT and MRI rather than the patch/tile/whole-slide settings of digital pathology. Ref. [29] is yet another recent work, but features mortality prediction tasks (often using EHR or clinical data), not image classification in histopathology. By contrast, the present systematic review specifically targets histopathology image classification using federated learning, thereby addressing a clear gap in the existing literature. None of the prior systematic reviews have provided a focused examination of this particular intersection of domain (histopathology), task (image classification), and methodology (federated learning). This review aims to bridge that gap by comprehensively synthesizing existing studies in this emerging field, analyzing the methodologies employed, datasets utilized, and the unique challenges encountered. Table 1 summarizes the key contributions and the histopathology coverage of previous survey studies.

## 3. Methodology

This section outlines the systematic approach used to conduct the literature review, following the PRISMA (Preferred Reporting Items for Systematic Reviews and Meta-Analyses) guidelines. The methodology includes the literature search process, selection criteria, and data extraction strategies to ensure a comprehensive and unbiased review of federated learning applications in histopathology image classification. In accordance with PRISMA reporting, we acknowledge that no prospective protocol registration was undertaken for this review.

### 3.1. Eligibility Criteria

In this section, we outline the inclusion and exclusion criteria applied to select studies for this review. The studies were grouped for synthesis based on key methodological and analytical similarities, such as dataset type, deep learning architecture, and performance metrics, allowing for a structured comparison of model performance across different designs. To ensure the relevance and quality of included studies, the selection was guided by the following criteria.

#### 3.1.1. Inclusion Criteria

Studies were included if they met the following conditions:

- Focused on histopathology image classification using histopathology datasets.
- Involved the development or application of deep learning models within a federated learning framework.
- Provided performance analysis or comparative evaluation of FL models.
- Published between 2020 and 2025.

#### 3.1.2. Exclusion Criteria

Studies were excluded based on the following criteria:

- Did not involve federated learning for histopathology image classification.
- Focused on non-healthcare domains or irrelevant medical applications.
- Inaccessible full-text articles.
- Duplicate records identified across multiple databases.
- Studies that used federated learning for cancer classification on datasets other than histopathology images.

### 3.2. Information Sources

We conducted a comprehensive search across multiple academic databases, including ScienceDirect, IEEE Xplore, MDPI, arXiv, PubMed, and Springer Nature Link. These databases were selected based on their relevance to prior research in deep learning, medical imaging, and healthcare technology, ensuring a thorough coverage of pertinent studies for this review.

### 3.3. Search Strategy

Although our search strategy covered studies from 2017, when federated learning (FL) was first introduced, our inclusion criteria were restricted to papers addressing the classification of histopathology images using federated learning. While federated learning may have been applied earlier to other histopathology-related tasks such as detection, segmentation, or patch-level feature extraction, we found no peer-reviewed publications prior to 2020 that specifically reported classification results on histopathology datasets within a federated learning framework. The earliest eligible studies matching our criteria appeared in 2020, with the number of publications increasing steadily thereafter. This temporal limitation explains why our final dataset consists of 24 studies published between 2020 and 2025, representing, to our knowledge, the full body of literature to date on this specific application.

To improve the accuracy and relevance of our search results, we used a combination of targeted keywords and Boolean operators. The search strategy included variations and combinations of phrases such as “Histopathology Image Classification using federated learning” OR “federated learning for Histopathology Image Classification” OR “federated learning” AND “Histopathology” AND “Image Classification”. The search strategy was limited to titles, abstracts, and keywords to maintain focus on the most pertinent studies.

**Figure 5:**
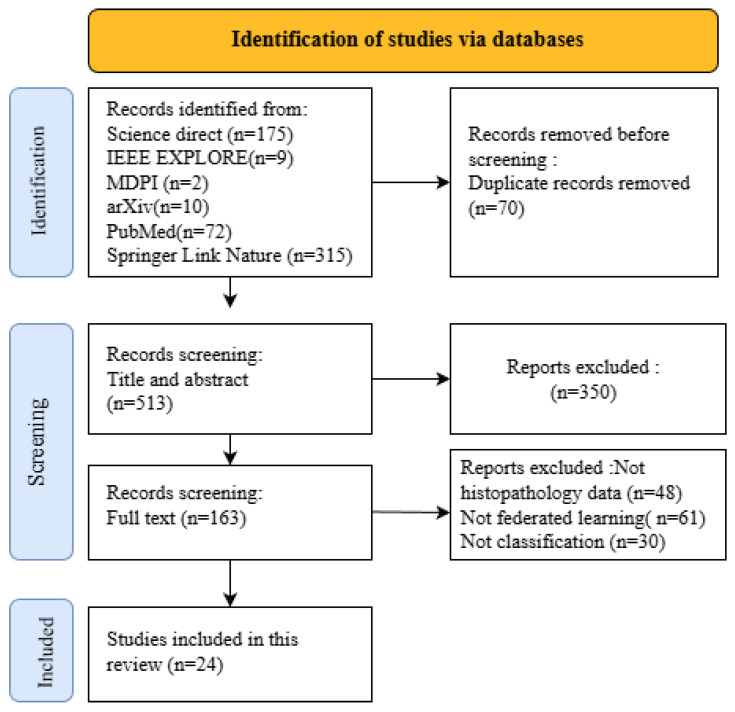
PRISMA (Preferred Reporting Items for Systematic Reviews and Meta Analyses) flow diagram illustrating the selection process of studies included in this systematic review.

The PRISMA flow diagram (Figure 5) illustrates the step-by-step process of identifying, screening, and selecting articles for inclusion in this review. This structured approach ensured a transparent and reproducible methodology.

### 3.4. Data Collection Process

Two reviewers independently screened all records and full-text reports to determine whether studies met the inclusion criteria for the review. Any disagreements were resolved through discussion to reach a consensus. No automation tools were used during the screening process.

### 3.5. Data Items and Extraction

Data were sought for key performance outcomes related to histopathological image classification, including accuracy, sensitivity, specificity, F1-score, and area under the receiver operating characteristic curve (AUC). For each outcome domain, we aimed to collect all compatible results reported in the studies, such as different evaluation metrics or analyses. In cases where multiple results were available for the same outcome (e.g., from various models or datasets within a study), selection was based on the most comprehensive and externally validated results to ensure consistency and comparability across studies. To support this process, we manually extracted key information from each study in a structured and consistent manner. This approach allowed for clear and systematic comparisons of findings across the diverse set of included studies. The data collected included the following:

- Bibliographic Details: Information such as the study title, authors, and year of publication.
- Datasets Used: Identification of histopathology datasets, including dataset source and size, to evaluate data diversity and generalizability.
- Classification Models and Federated Learning Frameworks: Documentation of deep learning architectures (e.g., CNNs, ResNet, and EfficientNet) and federated learning aggregation techniques (e.g., FedAvg, FedProx, and FedMA) used in the studies.
- Performance Metrics and Results: Extraction of performance indicators such as accuracy, precision, recall, specificity, F1-score, and AUC to assess model effectiveness.

This structured organization facilitated a clear comparative analysis, allowing us to identify trends, evaluate methodologies, and highlight gaps in the current literature.

### 3.6. Study Risk of Bias Assessment

Since our systematic review examines the use of machine learning and AI within the medical field, we found no existing risk-of-bias assessment tool suitable for this type of research. Consequently, following the methodology outlined in [30], we adopted a set of 12 evaluation questions to assess the quality of the included studies, as shown in Table 2. To operationalize this assessment, each criterion was scored using a three-level scale: Yes (1), Partially Yes (0), and No (−1). A score of Yes meant the study fully satisfied the criterion, Partially Yes indicated partial or unclear reporting, and No showed that the criterion was not covered. Each article was examined against all criteria.

### 3.7. Effect Measures

**Figure 6:**
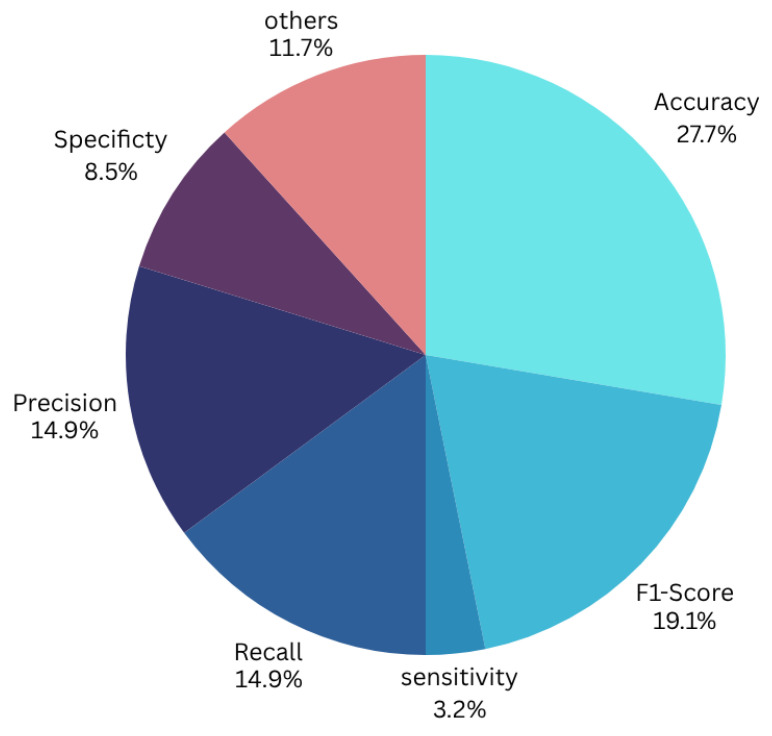
Performance evaluation of the models across different metrics.

The included studies employed a variety of evaluation metrics to assess model performance; however, accuracy was the most commonly reported metric across the majority of studies, as illustrated in Figure 6. Consequently, to enable a consistent and meaningful comparison of performance, this review primarily focuses on reporting and synthesizing accuracy results.

## 4. Result and Analysis

**Figure 7:**
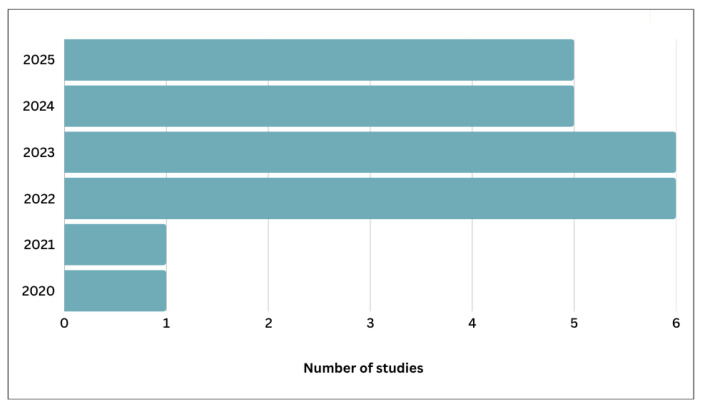
Growth pattern of research publications (2020–2025) focusing on federated learning approaches for histopathology image classification.

Following a comprehensive literature screening process, 24 eligible studies published between 2020 and 2025 were identified for inclusion in this review, Figure 7 shows growth pattern of research publications (2020–2025). These studies specifically examined the application of federated learning (FL) techniques for histopathology image classification. The publication trend indicates a clear growth in research activity beginning in 2022, with a peak in 2022 and 2023, during which six studies were published in each year. This represents a marked increase from one study in 2020 and one in 2021. The upward trend continued with five studies published in 2024 and another five in 2025, reflecting sustained and expanding interest in leveraging FL for digital pathology. This progression in publication output is illustrated in Figure 7.

### 4.1. Study Selection Process

Multiple phases were involved in selecting the studies, as presented in the PRISMA flow diagram (Figure 5). Initially, 583 articles were identified from the databases. A total of 163 papers underwent a full text examination after duplicates were eliminated and titles and abstracts were screened. Based on the predefined inclusion and exclusion criteria, 24 studies were ultimately included in the final analysis.

By applying rigorous selection and data extraction methods, the goal of this systematic review is to offer an in-depth overview of the current landscape of federated learning in histopathology image classification, highlighting both achievements and areas for future research.

### 4.2. Study Characteristics

Table 3 presents a summary of the key characteristics of the reviewed studies. It highlights the datasets used and the advantages and limitations of each study. This table serves as a comparative tool to highlight the effectiveness of different approaches and the challenges they address.

### 4.3. Reported Performance Comparison Between Centralized and Federated Training

A wide range of studies have compared federated learning (FL) with centralized learning (CL) for histopathology image classification, showing that FL often matches or surpasses centralized performance while preserving privacy. FedDropoutAvg [32] demonstrated improved F1 scores with increased client and parameter dropout, approaching centralized results, although its gains were most pronounced in settings with high client diversity, raising questions about its generalizability to more homogeneous cohorts. FedDBL [37] consistently outperformed other FL methods, especially in low-data regimes and even exceeded centralized training in certain settings, while reducing communication costs through one-round aggregation; however, its reliance on bidirectional knowledge distillation introduces additional hyperparameters that may complicate deployment. In [40], FL models achieved strong specificity and competitive recall/accuracy relative to centralized baselines, while [41] showed that multi-magnification fusion and attention improved performance further. These works underscore that architectural choices, rather than FL itself, often drive accuracy gains, complicating direct comparisons across studies.

HarmoFL [42] achieved stable and high accuracy across rounds, outperforming FedBN, though its batch-statistic harmonization assumes consistent domain shifts across silos, a condition not always met in real-world pathology networks. Similarly, ref. [43] confirmed FL robustness under limited label noise, but did not evaluate more challenging forms of annotation bias common in histopathology. Hybrid quantum FL [44] surpassed classical baselines with fewer parameters, yet its practical relevance remains constrained by currently limited quantum hardware. In contrast, ref. [45] showed that a federated consensus model outperformed both site-specific and centralized baselines, highlighting how ensembling across institutions can compensate for biases that centralized pooling may inadvertently amplify. SSL-FL-BT [46] provided consistent gains across datasets using semi-supervised techniques, though its reliance on large unlabeled sets may limit applicability. SiloBN [47] similarly outperformed FedAvg and FedProx while matching pooled training, emphasizing the importance of addressing distribution shift through normalization rather than aggregation rules alone.

Additional studies using MobileNet-v2 and DenseNet-201 [48], ensemble FL models such as YOLOv6 [49], and newer variants like FedImp [50], FedSAF [51], and FedWSIDD [52] reported accuracies close to centralized benchmarks, though many evaluated only balanced or moderately heterogeneous datasets, limiting conclusions about performance under severe inter-site variability. Other works likewise reported FL performance within 1–2% of centralized accuracy or Dice scores [53], while UniFed [54] offered substantial communication savings despite slightly lower accuracy, illustrating the ongoing trade-off between performance and efficiency across the FL design space.

**Figure 8:**
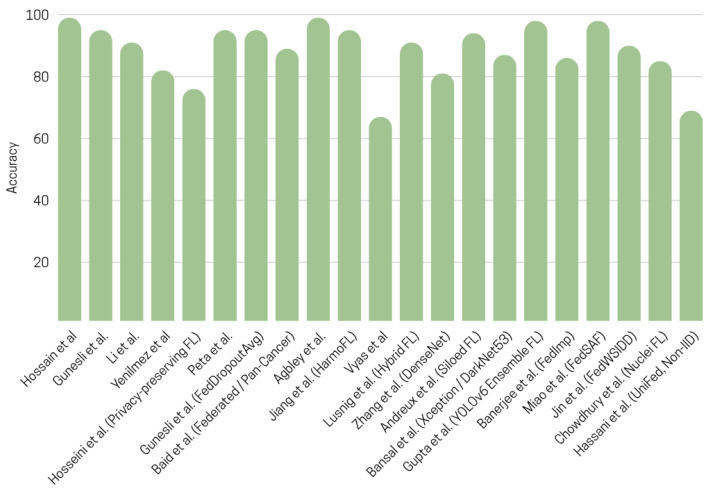
Accuracy comparison of federated learning approaches in histopathological image analysis across different studies [31,32,33,34,35,36,38,39,40,41,42,43,44,45,46,47,48,49,50,51,52,53,54].

Overall, although these studies collectively demonstrate that FL can achieve competitive or superior performance compared to centralized training while maintaining privacy, the methodological heterogeneity ranging from model architectures to dataset distributions, augmentation pipelines, and evaluation protocols complicates direct comparisons. Many approaches excel only under specific assumptions (e.g., moderate heterogeneity, availability of unlabeled data, or stable label quality), and relatively few works evaluate robustness across institutions with extreme domain shift. Figure 6 summarizes the distribution of performance metrics across studies, highlighting both the promise of FL and the need for more standardized benchmarking to enable fair and reproducible comparisons, and Figure 8 shows an accuracy comparison of federated learning approaches for histopathological image analysis across different studies.

### 4.4. Federated Learning Frameworks, Aggregation Strategies, and Architectures

Table 4 summarizes the key federated learning aggregation algorithms with brief definitions. Many studies in federated learning do not explicitly specify the frameworks they use; two widely discussed open source frameworks in the literature are OpenFL and Flower. These frameworks offer specialized tools and flexible architectures tailored to various federated learning applications, particularly in privacy-sensitive environments such as healthcare.

**OpenFL (Open federated learning)** is an open source software framework designed for federated learning (FL). It was initially developed by Intel Labs in collaboration with the University of Pennsylvania and is now maintained as a general-purpose framework for real-world applications [55]. OpenFL follows a star topology federated architecture composed of two node types: collaborators and a central aggregator. Each collaborator maintains its own local, private dataset, along with the federated-learning configuration, model definitions, and training procedures. Communication between the aggregator and collaborators occurs through gRPC connections secured with mutual TLS, allowing the aggregator to collect model updates and performance metrics without ever accessing raw data. The entire federation is coordinated by an FL plan, a YAML specification shared across all participants that outlines the tasks to execute, workflow steps, aggregation strategy, network endpoints, and the total number of training rounds. On each collaborator, the OpenFL runtime carries out whatever operations the plan specifies, such as training, evaluating, and submitting updated model parameters, while the aggregator manages the overall process and combines the updates into a refreshed global model before sending it back for additional rounds. The framework’s design also features a clearly defined backend responsible for protected remote procedure calls, authentication via a public-key infrastructure, optional deployment inside trusted execution environments (like Intel SGX), and mechanisms for serializing models and metrics to uphold privacy and integrity throughout the federated-learning life cycle [55].

**Flower (a friendly federated fearning framework)** is an open source, flexible, and scalable federated learning (FL) framework that allows the training of machine learning models across many decentralized devices or clients without requiring them to share their raw data. Flower’s design revolves around the interaction between global server-side coordination and local client-side computation, tied together through a protocol-centric and framework-independent architecture [56]. On the server, the main elements include the ClientManager, which maintains active clients through ClientProxy objects, the federated learning loop, and a modular Strategy component responsible for decisions such as choosing clients, setting round configurations, and aggregating updates. During each round, the server asks the Strategy for configuration details, dispatches instructions to selected clients, gathers their results or errors, and then hands the aggregation back to the Strategy. Clients operate via either the Edge Client Engine (for real hardware) or the Virtual Client Engine (VCE) for large-scale simulations. They receive protocol messages, run user-defined training or evaluation code, and return serialized outputs in a format independent of language or ML framework [56]. Flower’s architecture remains communication-agnostic through the abstract ClientProxy layer, enabling integration of diverse devices and transport mechanisms, while the VCE provides scalable virtualization and efficient scheduling for large numbers of clients. This framework was adopted in [36,44].

Following the examination of the federated learning frameworks used across the studies, the reviewed studies collectively highlight a diverse landscape of aggregation strategies in federated learning, ranging from classical averaging methods to more specialized, domain-adaptive approaches. While many works build on the simplicity and effectiveness of FedAvg, others introduce extensions designed to improve robustness under non-IID conditions, reduce communication overhead, or enhance privacy. More advanced algorithms incorporate weighting schemes and secure multi-party computation, attention mechanisms, or data-driven harmonization to address the challenges of institutional variability and heterogeneous feature distributions. Together, these methods reflect an evolving progression from general-purpose aggregation toward increasingly task-specific and context-aware solutions. Table 4 provides a concise summary of the aggregation algorithms used across all included studies.

Building on the discussion of aggregation algorithms, the studies reviewed employ a wide range of classification architectures, reflecting differing priorities in accuracy, efficiency, and adaptability to federated learning constraints. Deep convolutional networks dominate the landscape due to their strong feature extraction capabilities, while lighter architectures and transformer-based or MIL-based models appear in settings requiring scalability or whole-slide analysis. Despite architectural diversity, a consistent trend emerges: model effectiveness in federated settings is strongly tied to how well the chosen architecture balances representational power with computational feasibility across heterogeneous clients.

### 4.5. Staining Techniques and Datasets

The datasets used in this study consist primarily of Hematoxylin and Eosin (H&E) stained histopathological images, the standard modality for visualizing tissue morphology. H&E staining provides strong contrast between nuclei and surrounding tissue, making it well suited for distinguishing between benign and malignant patterns and for training deep learning models.

**Figure 9:**
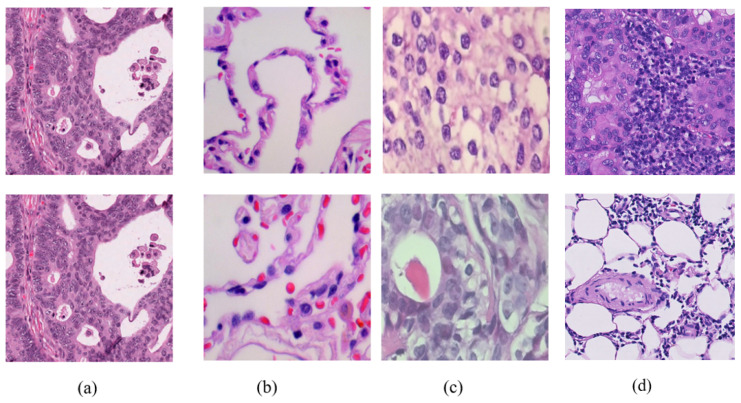
Sample images from various datasets used in the literature: (**a**) TCGA CRC-DX Dataset, (**b**) LC25000 Dataset, (**c**) BreakHis Dataset, and (**d**) Breast Histopathology Image (BHI).

Commonly used datasets across the reviewed studies include LC25000, TCGA, BreakHis, MC-CRC, and Camelyon16/17, covering various cancer types such as lung, colon, breast, and liver. These datasets supply the high-quality H&E images needed for model development, evaluation, and benchmarking in federated learning applications for histopathology image classification. Table 5 summarizes the key characteristics of the datasets, while Figure 9 presents representative images sourced from these datasets.

The reviewed studies employed a wide variety of histopathology datasets reflecting different imaging sources and diagnostic tasks. Commonly used datasets included BreakHis [33,36,41,48,49,50], offering breast tumor images at multiple magnifications; TCGA [32,35,38,39,45], providing multi-cancer whole slide images; HAM10000 for skin lesions [34,43]; Camelyon16/17 for breast cancer metastasis [42,47,52]; LC25000 [40] and MC-CRC [37] for colorectal and lung cancers; BHI for breast cancer patches [40]; PanNuke for nuclei segmentation [53]; and MedMNIST covering 2D and 3D biomedical images [54].

Some studies combined multiple datasets to address diverse tasks. For example, ref. [42] integrated Camelyon17, MoNuSAC, TNBC, and multi-site prostate MRI for segmentation, while [46] used four breast histopathology datasets for grading and tissue classification. Similarly, ref. [44] analyzed liver biopsies annotated across steatosis stages.

Overall, these datasets, ranging from single institution collections to multi-center cohorts, provide diverse, representative histopathological data that support robust model training, generalizability, and the evaluation of federated learning under realistic privacy and heterogeneity constraints.

### 4.6. Comparative Analysis of Results Across Reviewed Federated Learning Studies

Across the surveyed studies, notable differences emerge in how federated learning performs under varying data distributions, architectural choices, and aggregation strategies. Studies operating in more homogeneous or well-curated datasets generally reported stronger and more stable performance, whereas those conducted under highly non-IID or institutionally diverse conditions demonstrated greater sensitivity to algorithmic design and optimization techniques. Methods incorporating domain adaptation, privacy enhancements, or advanced aggregation strategies tended to achieve better generalization across sites, while frameworks evaluated on whole-slide images or complex multi-class tasks often faced additional challenges related to data scale and weak labeling. Despite these variations, a consistent theme across the literature is that federated learning maintains competitive performance relative to centralized baselines and in many cases improves robustness by leveraging distributed, institution-specific learning signals. The summarized results in Table 6 reflect these trends and highlight the strengths and limitations observed across different methodological directions.

### 4.7. Hardware and Software

The surveyed FL studies for histopathological image analysis employ diverse hardware and software setups. Reported configurations range from Google Colab with an NVIDIA A100 GPU (40 GB), 85 GB RAM, and five simulated clients [31], to local servers using RTX-series GPUs such as RTX 3090 with an Intel i9-11900K [37], RTX 2080 Ti in PyTorch-based pipelines [40], and similar RTX 2080 Ti systems in two-client FL settings [41]. Some works rely on PyTorch with Flower or PennyLane for hybrid quantum classical FL but omit hardware details [44], while others again use PyTorch on RTX 2080 Ti servers [46]. Across studies, Python and PyTorch dominate [53], with Adam (often LR = 0.0003) as a common optimizer and training organized into multiple local/global rounds (e.g., 10 × 5 or 50 local rounds) [50,52]. High-performance GPUs such as NVIDIA V100 and RTX models are frequently used [52,53], alongside Google Colab Pro with T4 GPUs [54]. Many studies incorporate pretrained models such as ImageNet initialized ResNet-50 to enhance convergence [52]. Overall, these configurations provide a reproducible and scalable foundation for FL experimentation across medical imaging tasks.

## 5. Discussion

Federated learning (FL) shows strong potential for privacy-preserving histopathology analysis, and its performance is shaped by several interconnected factors. Work on aggregation algorithms forms the core of this research: although FedAvg [8] is widely used, its sensitivity to non-IID data has motivated alternatives such as FedDropoutAvg [32,38], FedDBL [37], FedImp [50], SiloBN [47], and FedSAF [51], which offer improved robustness at the cost of additional complexity. Since aggregation performance is closely tied to the underlying model, many studies also evaluate how architecture influences outcomes. ResNet and DenseNet variants consistently perform well on datasets such as BreakHis [33,36,41,48,49,50] and TCGA [32,35,38,39,45], while MobileNet and EfficientNet [33,50] remain effective under resource constraints. Task-specific designs, such as MIL [52] and U-Net-based segmentation [53], further show how model choice aligns with the type of histopathological analysis. These architectural considerations naturally tie into data-related factors, as dataset diversity strongly affects generalization. Multi-center datasets like MC-CRC [37] and LC25000 [40] support broader robustness, while heterogeneous or single-source datasets [42,46] highlight the challenge of harmonization.

Performance in FL also depends on practical, system-level constraints. Communication bottlenecks, hardware heterogeneity, and pre-processing demands are well-documented limitations in medical imaging FL [31,37,40,41], motivating more communication-efficient and resilient aggregation protocols [11,26]. Because histopathology workflows involve substantial local pre-processing, studies emphasize the impact of tile extraction, stain normalization, and feature computation on overall training speed [50,52,53]. This situates data-handling efficiency as a central performance factor rather than a peripheral concern.

Taken together, the literature depicts a shift toward more adaptive and context-aware FL methods [8,31,33], illustrated by algorithms such as FedDropoutAvg, FedImp, FedSAF, HarmoFL, and cluster-based SMC [32,35,42,50,51]. At the same time, work by [34,39,40] shows that conventional or lightweight architectures can still perform strongly when distributional shifts and communication constraints are managed effectively. Remaining gaps include limited dataset diversity, over-reliance on simulated federation, and insufficient evaluation of extreme heterogeneity, client dropout, scalability, and fairness [52,54], along with inconsistent evaluation practices [43,47]. Complementary studies also show that data gathering and transformation can directly improve FL performance through multi-magnification fusion [41], stain-normalized synthetic slide distillation [52], broad learning heads [37], frequency-domain harmonization [42], and classical augmentation, feature selection (RGW/RDE), or imbalance handling (SMOTE) as discussed by [36]. Together, these works indicate that progress in FL for histopathology depends on coordinated improvements in aggregation, model design, data handling, and system efficiency. The quality assessment scores for each reviewed study are reported in Table 7.

### Limitations and Future Work

Federated learning in medical imaging remains constrained by communication overhead, hardware heterogeneity, and non-IID or imbalanced data, all of which hinder scalability and model generalization. Addressing these issues requires more communication-efficient protocols, stronger aggregation methods, and algorithms that handle heterogeneous and uneven data distributions. Future work in histopathology should focus on personalized and domain adaptation FL, model compression and asynchronous updates, privacy methods with minimal accuracy loss, and clinically oriented explainability. Establishing large-scale, standardized FL benchmarks is also essential for improving comparability and supporting real-world deployment.

## 6. Conclusions

In this systematic literature review, we examined research studies that implemented federated learning for histopathology image classification. Our analysis covers the datasets used, the frameworks utilized, their performance outcomes, and the classification models applied. This review aims to provide a comprehensive understanding of how federated learning contributes to overcoming data sharing limitations while maintaining robust classification performance in healthcare applications.

The classification of histopathology images using federated learning demonstrates strong potential across a wide range of applications and datasets, showcasing both high accuracy and robustness in the handling of data heterogeneity. Several studies highlight the ability of federated learning to achieve competitive performance compared to centralized models, with accuracies often exceeding 90% and, in some cases, close to 99%. The use of different models, such as VGG16, DenseNet, and ResNet, contributes significantly to these results, as they leverage the power of deep learning to process complex medical imaging tasks effectively.

## Figures and Tables

**Table 1:** Comparison of key aspects, findings, and contributions between existing review studies and the present review.

Study	Domain Focus	Key Contributions/Findings	Coverage of Histopathology
Nazir et al. (2023) [11]	Medical image FL (classification and segmentation)	Reviews FL for general medical imaging; covers architectures and performance.	Does not address histopathology tile-based classification.
Prayitno et al. (2021) [24]	General healthcare FL	Surveys FL across healthcare; discusses data partitioning, data distribution, and COVID-19 imaging.	Very limited; histopathology barely mentioned.
Teo et al. (2024) [25]	FL in healthcare (612 studies)	Finds only 5.2% of studies are real-world; radiology dominates; pathology/histology underrepresented.	Limited attention to pathology/histology.
Yang et al. (2024) [26]	FL challenges in medical image analysis	Discusses heterogeneity, label quality, and technical challenges.	Only peripheral mention of histopathology.
Sandhu et al. (2023) [27]	Medical FL categorized by disease, modality, body part	Gives broad overview of FL architectures and performance vs. traditional ML.	No dedicated section on histopathology classification.
Raza et al. (2025) [28]	Radiomics and medical image FL	Focuses on CT, MRI, PET radiomics workflows.	Radiomics usually excludes histopathology tile/WSI analysis.
Tahir et al. (2025) [29]	Healthcare FL for mortality prediction	Covers EHR and clinical prediction tasks, not imaging.	No coverage of histopathology image classification.
This review (2025)	Surveys FL for histopathology image classification	outlines tissue-level variability, annotation considerations, and diagnostic challenges in digital pathology.	Focuses exclusively on histopathology images; no other imaging modalities are considered

**Table 2:** Quality assessment questions used for evaluating the included studies.

Question Label	Question
Q1	Is the aim of the research stated clearly?
Q2	Is the size of the dataset adequate for this type of analysis?
Q3	Is the procedure for managing data in the federated setting described in detail?
Q4	Does the study address the issue of non-IID data distribution?
Q5	Are any additional privacy-preserving techniques implemented?
Q6	Does the author offer enough information about the experimental setup?
Q7	Are the learning methods explained thoroughly?
Q8	Are the outcomes of the study presented clearly?
Q9	Is there a comparison between the different methods or approaches used?
Q10	Are the study’s limitations acknowledged?
Q11	Does the research contribute meaningfully to the existing body of literature?
Q12	Does the study make any tools or source code available online?

**Table 3:** Summary of existing studies highlighting their advantages, limitations, and datasets used in federated learning for medical applications.

Ref	Author	Dataset	Advantages	Limitations
[31]	Hossain et al.	LC2500 dataset	Explainable AI Integration, Decentralized Training Improved Generalization	IID Assumption, Computational Complexity, Data Heterogeneity
[32]	Gozde N. Gunesli et al.	TCGA CRC-DX Dataset	Handles Data Heterogeneity, Reduces Overfitting, Performs Well on Unseen Data	Potential for Model Bias, Client Dropout Effects Computational Complexity
[33]	Lingxiao Li et al.	BreakHis Dataset	Effective Knowledge Fusion Comparable Performance to Centralized Learning, Supports Multi-Client Training	Encryption-Related Efficiency, Issues Lack of Large-Scale Validation, Potential Performance Drop in Non-IID Settings
[34]	Musa Yenilmez et al.	HAM10000 Dataset	Uses Pre-Trained Models for Efficiency Handles Multi-Client Scenarios Ensures Data Privacy	Limited to Specific Skin Lesions Potential Model Performance Drop Lack of Advanced Aggregation Techniques
[35]	S. Maryam Hosseini et al.	The Cancer Genome Atlas (TCGA) Dataset	Enhanced Privacy Protection Maintains Model Accuracy Cluster-Based Model Aggregation	Higher Communication Overhead Scalability Concerns Computational Complexity
[36]	Jyothi Peta et al.	BreakHis Dataset	Secure Image Transmission, Optimal Key Generation, High Classification Performance	Potential Performance, Drop in Non-IID Scenarios Encryption Complexity, Higher Communication Costs
[37]	Tianpeng Deng et al.	Multicenter Colorectal Cancer (MC-CRC) Dataset	Highly Communication-Efficient Performs Well with Limited Training Samples, Model Generalization to External Datasets	Performance Can Be Affected by Data Heterogeneity, Limited to Pretrained Deep Learning Backbones, Encryption Overhead
[38]	Gozde N. Gunesli et al.	TCGA CRC-DX Dataset	Handles Data Heterogeneity, Reduces Overfitting, Increases Model Robustness	Not Ideal for Small-Scale Studies Potential Model Convergence Challenges Communication Efficiency Not Fully Optimized
[39]	Ujjwal Baid et al.	The Cancer Genome Atlas (TCGA) Dataset	Handles Out of Distribution Data, Consensus Model, Achieves High Accuracy, Efficient Federated Training Framework	Slower Convergence, Limited to a Single Network Architecture, Data Heterogeneity Issues
[40]	Y. Agbley et al.	LC25000 Dataset	Preserves Data Privacy, Improves Model Generalization Handles Multi-Class Classification	Communication Overhead Computationally Intensive Data Heterogeneity Issues
[41]	Y. Agbley et al.	BreakHis Dataset	Multi-Magnification Image Fusion, Self-Attention Mechanism High Classification Performance	Computationally Intensive, Higher Storage Requirements, Communication Overhead in FL
[42]	Meirui Jiang et al.	Camelyon17 Dataset	Reduces Local and Global Model Drift, Improves Federated Model Convergence, Minimizes Communication Overhead	Requires Frequency-Domain Transformations, Limited to Certain Medical Imaging Tasks
[43]	Sankalp Vyas et al.	HAM10000 Dataset	Privacy-Preserving Learning, Protects Against Data Poisoning Attacks, Decentralized Model Training	Vulnerable to Malicious Clients Computational Overhead, Higher Communication Costs
[44]	Luca Lusnig et al.	Liver Biopsy Image Dataset	Hybrid Quantum Neural Network (HQNN) for Feature Learning Superior Classification Accuracy Efficient Learning on Small Datasets	Limited Quantum Hardware Availability, Potential Performance Drop in Highly Heterogeneous Data, Federated Learning Communication Overhead
[45]	Ujjwal Baid et al.	The Cancer Genome Atlas (TCGA) Dataset	Preserves Data Privacy, Improves Generalization Across Cancer Types, Comparable to Centralized Training	Data Heterogeneity, Challenges Computational Complexity, Limited Evaluation on Other Architectures
[46]	Yuanming Zhang et al.	2015 Bioimaging Challenge Dataset 4th Symposium in Applied Bioimaging Dataset ICIAR 2018 Grand Challenge on Breast Cancer Histology Images Dataset Databiox Dataset	Multi Task Self Supervised Learning, Handles Non-IID Data in FL Contrastive Learning for Robust Training	Computationally Intensive Requires High-Quality Pseudo Data Higher Communication Costs in FL
[47]	Mathieu Andreux et al.	Camelyon16 and Camelyon17 Datasets	Improves Generalization Across Institutions, Enhances Privacy Protection Maintains High Performance on Non-IID Data	Increased Computational Overhead, Not Ideal for Small Datasets, Higher Communication Costs in Federated Learning
[48]	Shubhansh Bansal	BreakHis Dataset	Transfer Learning with Pretrained Models, Handles Data Imbalance with Balanced Accuracy, High Classification Accuracy	Computational Complexity, Vulnerability to Non-IID Data, Communication Overhead in Federated Learning
[49]	Chhaya Gupta et al.	BreakHis Dataset	Data privacy preservation, High accuracy, Reduced communication overhead, Model compression, Enhanced generalization, Efficient training	Non-IID data challenges, Security vulnerabilities, Data imbalance issues, Communication costs & scalability
[50]	Mangaldeep Banerjee et al.	BreakHis1, BreakHis2, BRACS1, BRACS2	Enhanced performance, Handles data heterogeneity, Maintains model consistency, Focus on important layers, Privacy preservatio	Lack of explicit data distribution utilization, Potential computational overhead, Limited task scope
[51]	Yuxin Miao et al.	SEED, BOT (gastric cancer histopathology)	Enhanced model personalization, Improved communication efficiency, Robustness to data heterogeneity, Higher accuracy and generalization	Limited model architecture flexibility, Synchronous update constraints, Potential overfitting with high personalization
[52]	Haolong Jin et al.	CAMELYON16, CAMELYON17	Model flexibility, Reduced communication cost, Improved performance, Stain normalization integration, Rapid convergence, Enhanced generalization	Synthetic data explainability issues, High computational cost of distillation, Hyperparameter sensitivity, Limited variability in synthetic data, Performance drop without stain normalization
[53]	AnjirChowdhury et al.	PanNuke dataset	Joint segmentation and classification Context-aware learning, Handles overlapping nuclei	High annotation requirement, High computational cost, Generalizability issues, Error propagation, Challenges with small structures, Clinical adoption barriers
[54]	Atefe Hassani et al.	TissueMNIST (MedMNIST2D)	Handles high heterogeneity, Dynamic training strategy, Curriculum learning integration, Efficient communication, Improved convergence time, Better performance	Increased system complexity, Higher initial computation, Dependence on task complexity estimation, Sequential dependency

**Table 4:** Summary of key federated learning aggregation algorithms, including concise definitions of each method.

Aggregation Algorithm	Description
FedAvg	Traditional federated averaging method that computes a weighted average of client model updates.
FedDropoutAvg	Extends FedAvg by applying dropout at model and client levels to improve robustness and reduce overfitting.
FedDBL	Uses weighted averaging to optimize communication efficiency and performance with limited training samples.
FedImp	Emphasizes the importance of specific model layers during aggregation using a semantically weighted strategy, reducing deviation between client and global models.
FedImpAvg	Refines FedImp by performing weighted averaging based on the number of local training samples.
Cluster-Based SMC	Secure aggregation by distributing model updates within small clusters before global aggregation to enhance privacy.
SiloBN	Retains local batch normalization statistics while sharing only learned BN parameters, improving model adaptation across clients.
FedSAF	Integrates Attention Message Passing (AMP) with the Fisher Information Matrix (FIM) to dynamically adjust client contributions based on model similarity.
FedWSIDD	Dataset distillation based aggregation where clients generate synthetic slides that are aggregated and redistributed to enhance generalization.
Dynamic Sequential Aggregation	Orders clients based on task complexity and sequentially updates the global model, improving convergence and communication efficiency.

**Table 5:** Summary of key characteristics for the datasets used in the literature.

Ref	Dataset Name	Domain/Modality	Task	Samples	Source	Paper & Approach
[57]	The BreakHis dataset	Histopathology	Classification	7909 microscopic images of breast tumor tissue from 82 patients	Public	[33,36,41,48] Horizontal FL
[58]	The TCGA (The Cancer Genome Atlas)dataset	Histopathology	Classification	contains 512 × 512 non-overlapping tiles from WSIs of colorectal cancer (CRC) across 36 institutions	Public	[32,35] Horizontal FL, [39,45] Vertical FL
[59]	The HAM10000 dataset	Histopathology	Classification	10,015 dermoscopic images of seven different skin lesion types	Public	[34,43] Horizontal FL
[60]	The Camelyon dataset	Histopathology	Classification	170 WSIs (100 normal, 70 with metastases) 100 WSIs (60 normal, 40 with metastases)	Public	[42,47] Horizontal FL
[61]	The LC25000 dataset	Histopathology	Classification	25,000 histopathology images of lung and colon cancer biopsies	Public	[31,40] Horizontal FL
-	The Multicenter CRC (MC-CRC)	Histopathology	Classification	includes colorectal cancer histopathology images with nine tissue classes	Public	[37] Horizontal FL
-	Breast Histopathology Image (BHI)	Histopathology	Classification	Contains 277,524 histopathological image patches at 40× magnification	-	[40] Horizontal FL
-	Liver Biopsy Image Dataset	Histopathology	Classification	Contains 4400 histopathological liver biopsy images	An anonymous teaching archive at the University of Trieste	[44] Horizontal FL
[62]	PanNuke dataset	Histopathology	Classification and segmentation	includes 205,343 annotated nuclei, each accompanied by an instance segmentation mask.	Public	[53]
[63]	MedMNIST	Histopathology	Classification	a large-scale MNIST-like collection of standardized biomedical images, including 12 datasets for 2D and 6 datasets for 3D.	Public	[54]

**Table 6:** Overview of the aggregation strategies, classification architectures, and performance outcomes reported across the reviewed studies.

Paper	Aggregation	Classification Models	Results
Hossain et al., 2024 [31]	FedAvg	Inception-V3	Near-perfect: 99.867% (lung), 100% (colon), 99.720% (combined)
Güneşli et al., 2023/2021 [32,38]	FedDropoutAvg	ResNet18 + GroupNorm	AUC 0.965 (local), AUC 0.954 (independent); mean F1 = 0.9102, AUC = 0.9542
Li et al., 2022 [33]	FedAvg	ResNet-512, DenseNet-201, MobileNet-v2-100, EfficientNet-b7	Image-level ACC (ACCIL): 84.02–91.06%; Patient-level ACC (ACCPL): 84.09–91.87%
Yenilmez et al., 2024 [34]	FedAvg	VGG16	82.04% accuracy
Peta et al., 2023 [36]	FedAvg	C2T2Net	95.68% accuracy; “all key metrics > 95%”
Baid et al., 2022/2024 [39,45]	FedAvg	VGG16	Balanced accuracies around 89%
Agbley et al., 2022/2023 [40,41]	FedAvg	Hybrid (e.g., ResNet18, ResNet50, GaborNet); ResNet + self-attention	Very high: 99.87% and 99.99% (lung), 99.72% (colon).
Vyas et al., 2023 [43]	FedAvg		67.1% accuracy
Lüsnig et al., 2024 [44]	FedAvg	ResNet-512, Hybrid Quantum ResNet (QDI layers)	91.06% accuracy
Zhang et al., 2023 [46]	FedAvg	ResNet-50	81.48% accuracy (DenseNet)
Bansal et al., 2023 [48]	FedAvg	Xception, DarkNet53	Balanced accuracy: Xception 83.07%, DarkNet53 87.17%
Gupta et al., 2025 [49]	FedAvg	YOLOv6	98% (BreakHis), 97% (BUSI); Recall 99%, F1 98%
Chowdhury et al., 2025 [53]	FedAvg	U-Net-style	84–85% accuracy (segmentation/classification)
Andreux et al., 2020 [47]	SiloBN	Batch-normalized DCNNs	Mean accuracy = 0.94
Hosseini et al., 2022 [35]	Cluster-based SMC	MIL gated attention classifier	76.65% (F1 = 80.48%) and 76.16% (F1 = 79.84%) (privacy-preserving settings)
Miao et al., 2025 [51]	FedSAF	Various comparative CNNs (AlexNet, ResNet18, EfficientNet-B0, MobileNetV3 Small)	98.43% (SEED dataset) and 81.16% (BOT dataset)
Jin et al., 2025 [52]	FedWSIDD	MIL methods for WSI: CLAM, TransMIL, ABMIL	90.1% ± 0.2 (CAMELYON16), 81.2% ± 1.2 (CAMELYON17)
Banerjee et al., 2025 [50]	FedImp/FedImpAvg	Efficient/modern nets referenced (e.g., EfficientNet-B3/B7 & EfficientViT)	Accuracy = 0.86; AUROC: 0.86 ± 0.08 (BreakHis1), 0.80 ± 0.01 (BRACS1)
Jiang et al., 2022 [42]	HarmoFL	DenseNet-201	95.48% accuracy
Hassani et al. [54]	UniFed	small CNNs (2-layer CNN+FFN), VGG11, ResNet18	69.37% (strongly non-IID) vs. 77.10% centralized; outperformed FedAvg (38.44%) and FedProx (37.92%)
T. Deng et al. [37]	FedDBL	EfficientNet/variants	92.13%

**Table 7:** Assessment criteria and the ratings obtained by the studies.

Ref	Q1	Q2	Q3	Q4	Q5	Q6	Q7	Q8	Q9	Q10	Q11	Q12	Score
[31]	1	1	1	−1	−1	1	1	1	1	1	1	−1	6
[32]	1	1	1	1	−1	1	1	1	1	0	1	−1	7
[33]	1	0	0	1	1	1	1	1	1	0	1	−1	7
[34]	1	1	1	−1	−1	1	1	1	1	−1	1	−1	4
[35]	1	1	1	1	1	1	1	1	1	0	1	−1	9
[36]	1	1	0	1	1	1	1	1	1	0	1	-−1	8
[37]	1	1	1	0	1	1	1	1	1	0	1	1	9
[38]	1	1	1	0	−1	1	1	1	1	0	1	−1	6
[39]	1	1	1	1	−1	1	1	1	1	1	1	−1	8
[40]	1	1	0	−1	−1	1	1	1	1	−1	1	−1	4
[45]	1	0	1	1	−1	0	1	1	0	−1	1	−1	3
[46]	1	0	1	0	1	0	1	0	0	0	1	−1	4
[47]	1	0	1	0	−1	1	1	1	1	0	1	−1	5
[48]	1	1	1	1	−1	0	0	1	1	0	1	−1	5
[42]	1	1	1	1	−1	0	0	1	1	0	1	1	9
[41]	1	1	1	0	−1	1	1	1	0	0	1	−1	5
[43]	1	1	1	−1	−1	1	1	1	0	0	1	−1	4
[49]	1	1	1	1	1	1	1	1	1	1	1	−1	10
[50]	1	0	1	1	−1	1	1	1	1	0	1	1	8
[51]	1	0	1	1	−1	0	1	1	0	0	1	−1	4
[52]	1	1	1	1	0	1	1	1	1	0	1	−1	8
[53]	1	1	0	0	−1	1	1	1	1	0	1	−1	5
[54]	1	0	1	1	−1	1	1	1	1	0	1	1	8

## Data Availability

This study is a systematic review and did not generate or analyze new datasets. All datasets referenced can be accessed through the original studies cited in the reference list.
